# Ethyl 3-[1-(4-bromo­phen­yl)eth­ylidene]carbazate

**DOI:** 10.1107/S1600536809044614

**Published:** 2009-10-31

**Authors:** Yu-Feng Li, Hai-Xing Liu, Fang-Fang Jian

**Affiliations:** a Microscale Science Institute, Department of Chemistry and Chemical Engineering, Weifang University, Weifang 261061, People’s Republic of China; b Microscale Science Institute, Weifang University, Weifang 261061, People’s Republic of China

## Abstract

In the crystal of the title compound, C_10_H_11_BrN_2_O_2_, the mol­ecules are linked by N—H⋯O hydrogen bonds, forming *S*(4) chains propagating in [100]. A C—H⋯O inter­action also occurs.

## Related literature

For background to Schiff bases, see: Cimerman *et al.* (1997 ▶). 
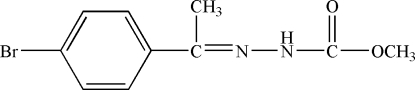


## Experimental

### 

#### Crystal data

C_10_H_11_BrN_2_O_2_
*M* *_r_* = 271.11
Orthorhombic, 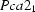

*a* = 7.6810 (15) Å
*b* = 5.9520 (12) Å
*c* = 24.750 (5) Å
*V* = 1131.5 (4) Å^3^
*Z* = 4
Mo *K*α radiation
μ = 3.62 mm^−1^
*T* = 293 K
0.25 × 0.20 × 0.19 mm

#### Data collection

Bruker SMART CCD diffractometer
Absorption correction: none
10040 measured reflections
2583 independent reflections
1675 reflections with *I* > 2σ(*I*)
*R*_int_ = 0.116

#### Refinement

*R*[*F* ^2^ > 2σ(*F* ^2^)] = 0.052
*wR*(*F* ^2^) = 0.152
*S* = 1.02
2583 reflections
140 parameters
1 restraint
H atoms treated by a mixture of independent and constrained refinement
Δρ_max_ = 0.45 e Å^−3^
Δρ_min_ = −0.54 e Å^−3^
Absolute structure: Flack (1983 ▶), 1254 Friedel pairs
Flack parameter: −0.008 (16)

### 

Data collection: *SMART* (Bruker, 1997 ▶); cell refinement: *SAINT* (Bruker, 1997 ▶); data reduction: *SAINT*; program(s) used to solve structure: *SHELXS97* (Sheldrick, 2008 ▶); program(s) used to refine structure: *SHELXL97* (Sheldrick, 2008 ▶); molecular graphics: *SHELXTL* (Sheldrick, 2008 ▶); software used to prepare material for publication: *SHELXTL*.

## Supplementary Material

Crystal structure: contains datablocks global, I. DOI: 10.1107/S1600536809044614/hb5186sup1.cif

Structure factors: contains datablocks I. DOI: 10.1107/S1600536809044614/hb5186Isup2.hkl

Additional supplementary materials:  crystallographic information; 3D view; checkCIF report

## Figures and Tables

**Table 1:** Hydrogen-bond geometry (Å, °)

*D*—H⋯*A*	*D*—H	H⋯*A*	*D*⋯*A*	*D*—H⋯*A*
N1—H1*D*⋯O1^i^	0.76 (10)	2.26 (10)	2.929 (6)	148 (10)
C3—H3*A*⋯O1^i^	0.96	2.43	3.242 (8)	142

Symmetry code: (i) 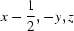.

**Fig. 1.**
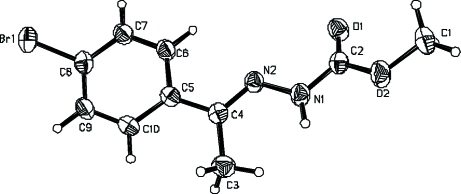
The structure of (I) showing 30% probability displacement ellipsoids.

## supplementary crystallographic information

### Experimental

A mixture of 1-(4-bromophenyl)ethanone (0.1 mol), and methyl carbazate (0.1 mol) was stirred in refluxing ethanol (20 mL) for 4 h to afford the title compound (0.085 mol, yield 85%). Colourless blocks of (I) were obtained by recrystallization from ethanol at room temperature.

### Refinement

The N-bound H atom was located in a difference map and freely refined. The other H atoms were fixed geometrically and allowed to ride on their attached atoms, with C—H distances=0.97 Å, and with *U*_iso_=1.2–1.5*U*_eq_.

### Crystal data

**Table:**

C_10_H_11_BrN_2_O_2_	*D*_x_ = 1.591 Mg m^−^^3^
*M**_r_* = 271.11	Mo *K*α radiation, λ = 0.71073 Å
Orthorhombic, *P**c**a*2_1_	Cell parameters from 1982 reflections
*a* = 7.6810 (15) Å	θ = 3.6–27.5°
*b* = 5.9520 (12) Å	µ = 3.62 mm^−^^1^
*c* = 24.750 (5) Å	*T* = 293 K
*V* = 1131.5 (4) Å^3^	Block, colourless
*Z* = 4	0.25 × 0.20 × 0.19 mm
*F*(000) = 544	

### Data collection

**Table:**

Bruker SMART CCD diffractometer	1675 reflections with *I* > 2σ(*I*)
Radiation source: fine-focus sealed tube	*R*_int_ = 0.116
graphite	θ_max_ = 27.5°, θ_min_ = 3.3°
ω scans	*h* = −9→8
10040 measured reflections	*k* = −7→7
2583 independent reflections	*l* = −31→32

### Refinement

**Table:**

Refinement on *F*^2^	Secondary atom site location: difference Fourier map
Least-squares matrix: full	Hydrogen site location: inferred from neighbouring sites
*R*[*F*^2^ > 2σ(*F*^2^)] = 0.052	H atoms treated by a mixture of independent and constrained refinement
*wR*(*F*^2^) = 0.152	*w* = 1/[σ^2^(*F*_o_^2^) + (0.068*P*)^2^] where *P* = (*F*_o_^2^ + 2*F*_c_^2^)/3
*S* = 1.01	(Δ/σ)_max_ = 0.054
2583 reflections	Δρ_max_ = 0.45 e Å^−^^3^
140 parameters	Δρ_min_ = −0.54 e Å^−^^3^
1 restraint	Absolute structure: Flack (1983), 1254 Friedel pairs
Primary atom site location: structure-invariant direct methods	Flack parameter: −0.008 (16)

### Special details

**Table:**

Geometry. All e.s.d.'s (except the e.s.d. in the dihedral angle between two l.s. planes) are estimated using the full covariance matrix. The cell e.s.d.'s are taken into account individually in the estimation of e.s.d.'s in distances, angles and torsion angles; correlations between e.s.d.'s in cell parameters are only used when they are defined by crystal symmetry. An approximate (isotropic) treatment of cell e.s.d.'s is used for estimating e.s.d.'s involving l.s. planes.
Refinement. Refinement of *F*^2^ against ALL reflections. The weighted *R*-factor *wR* and goodness of fit *S* are based on *F*^2^, conventional *R*-factors *R* are based on *F*, with *F* set to zero for negative *F*^2^. The threshold expression of *F*^2^ > σ(*F*^2^) is used only for calculating *R*-factors(gt) *etc*. and is not relevant to the choice of reflections for refinement. *R*-factors based on *F*^2^ are statistically about twice as large as those based on *F*, and *R*- factors based on ALL data will be even larger.

### Fractional atomic coordinates and isotropic or equivalent isotropic displacement parameters (Å^2^)

**Table:**

	*x*	*y*	*z*	*U*_iso_*/*U*_eq_	
Br1	0.44214 (8)	0.19577 (12)	0.58046 (2)	0.0815 (3)	
C8	0.3535 (7)	0.2162 (9)	0.5091 (2)	0.0584 (13)	
C1	0.0415 (10)	−0.3211 (12)	0.1630 (3)	0.080 (2)	
H1A	−0.0495	−0.3171	0.1364	0.120*	
H1B	0.0344	−0.4590	0.1830	0.120*	
H1C	0.1526	−0.3121	0.1454	0.120*	
N1	0.1035 (6)	0.0551 (8)	0.27043 (18)	0.0548 (10)	
C7	0.3861 (7)	0.0435 (10)	0.4736 (2)	0.0600 (12)	
H7A	0.4517	−0.0798	0.4845	0.072*	
C2	0.1408 (6)	−0.1185 (9)	0.23832 (19)	0.0515 (11)	
O2	0.0223 (6)	−0.1363 (9)	0.19887 (18)	0.0724 (12)	
C9	0.2600 (7)	0.4010 (9)	0.4932 (2)	0.0610 (12)	
H9A	0.2385	0.5177	0.5172	0.073*	
C4	0.1498 (6)	0.2404 (8)	0.3496 (2)	0.0463 (10)	
C5	0.2249 (6)	0.2378 (8)	0.4043 (2)	0.0470 (10)	
N2	0.1849 (5)	0.0686 (7)	0.31978 (17)	0.0509 (9)	
C10	0.1984 (6)	0.4113 (9)	0.4408 (2)	0.0542 (11)	
H10A	0.1374	0.5381	0.4297	0.065*	
C6	0.3215 (7)	0.0544 (9)	0.4220 (2)	0.0552 (12)	
H6A	0.3428	−0.0637	0.3983	0.066*	
O1	0.2632 (5)	−0.2456 (6)	0.24299 (17)	0.0611 (9)	
C3	0.0382 (7)	0.4343 (10)	0.3314 (3)	0.0633 (14)	
H3A	0.0001	0.4090	0.2950	0.095*	
H3B	0.1047	0.5707	0.3329	0.095*	
H3C	−0.0613	0.4469	0.3547	0.095*	
H1D	0.017 (13)	0.111 (17)	0.276 (4)	0.10 (3)*	

### Atomic displacement parameters (Å^2^)

**Table:**

	*U*^11^	*U*^22^	*U*^33^	*U*^12^	*U*^13^	*U*^23^
Br1	0.1019 (5)	0.0934 (5)	0.0491 (3)	−0.0009 (3)	−0.0070 (4)	−0.0089 (4)
C8	0.057 (3)	0.067 (3)	0.051 (3)	−0.003 (2)	0.007 (2)	−0.008 (2)
C1	0.099 (5)	0.077 (5)	0.063 (4)	−0.003 (3)	−0.014 (3)	−0.023 (3)
N1	0.049 (2)	0.066 (3)	0.050 (2)	0.004 (2)	−0.0056 (19)	−0.007 (2)
C7	0.063 (3)	0.060 (3)	0.057 (3)	0.010 (2)	−0.006 (2)	−0.008 (2)
C2	0.050 (3)	0.059 (3)	0.045 (2)	−0.006 (2)	−0.003 (2)	−0.003 (2)
O2	0.070 (2)	0.085 (3)	0.062 (3)	0.011 (2)	−0.024 (2)	−0.020 (2)
C9	0.073 (3)	0.055 (3)	0.056 (3)	0.004 (3)	0.008 (2)	−0.015 (3)
C4	0.040 (2)	0.051 (2)	0.049 (3)	−0.0062 (19)	0.0064 (19)	−0.001 (2)
C5	0.039 (2)	0.047 (2)	0.055 (3)	−0.0015 (18)	0.0064 (19)	−0.004 (2)
N2	0.048 (2)	0.057 (2)	0.047 (2)	0.0031 (17)	0.0013 (16)	−0.002 (2)
C10	0.055 (3)	0.045 (3)	0.063 (3)	0.0062 (19)	0.003 (2)	−0.007 (2)
C6	0.058 (3)	0.057 (3)	0.051 (3)	0.009 (2)	0.003 (2)	−0.009 (2)
O1	0.055 (2)	0.064 (2)	0.065 (2)	0.0031 (18)	−0.0094 (16)	−0.0121 (18)
C3	0.061 (3)	0.058 (3)	0.070 (4)	0.008 (2)	−0.007 (2)	−0.002 (3)

### Geometric parameters (Å, °)

**Table:**

Br1—C8	1.896 (6)	C2—O2	1.339 (6)
C8—C9	1.372 (8)	C9—C10	1.380 (7)
C8—C7	1.376 (7)	C9—H9A	0.9300
C1—O2	1.421 (8)	C4—N2	1.289 (6)
C1—H1A	0.9600	C4—C5	1.473 (7)
C1—H1B	0.9600	C4—C3	1.507 (7)
C1—H1C	0.9600	C5—C10	1.387 (7)
N1—C2	1.335 (7)	C5—C6	1.391 (7)
N1—N2	1.374 (6)	C10—H10A	0.9300
N1—H1D	0.75 (10)	C6—H6A	0.9300
C7—C6	1.371 (7)	C3—H3A	0.9600
C7—H7A	0.9300	C3—H3B	0.9600
C2—O1	1.212 (6)	C3—H3C	0.9600
			
C9—C8—C7	120.7 (5)	C10—C9—H9A	120.5
C9—C8—Br1	120.5 (4)	N2—C4—C5	115.8 (4)
C7—C8—Br1	118.8 (4)	N2—C4—C3	123.8 (5)
O2—C1—H1A	109.5	C5—C4—C3	120.4 (4)
O2—C1—H1B	109.5	C10—C5—C6	117.2 (5)
H1A—C1—H1B	109.5	C10—C5—C4	122.3 (4)
O2—C1—H1C	109.5	C6—C5—C4	120.5 (4)
H1A—C1—H1C	109.5	C4—N2—N1	117.4 (4)
H1B—C1—H1C	109.5	C9—C10—C5	121.9 (5)
C2—N1—N2	118.5 (4)	C9—C10—H10A	119.1
C2—N1—H1D	129 (7)	C5—C10—H10A	119.1
N2—N1—H1D	103 (7)	C7—C6—C5	121.6 (5)
C8—C7—C6	119.6 (5)	C7—C6—H6A	119.2
C8—C7—H7A	120.2	C5—C6—H6A	119.2
C6—C7—H7A	120.2	C4—C3—H3A	109.5
O1—C2—N1	126.4 (5)	C4—C3—H3B	109.5
O1—C2—O2	123.2 (5)	H3A—C3—H3B	109.5
N1—C2—O2	110.4 (5)	C4—C3—H3C	109.5
C2—O2—C1	116.5 (5)	H3A—C3—H3C	109.5
C8—C9—C10	119.0 (5)	H3B—C3—H3C	109.5
C8—C9—H9A	120.5		
			
C9—C8—C7—C6	−1.3 (8)	C3—C4—C5—C6	177.1 (5)
Br1—C8—C7—C6	179.3 (4)	C5—C4—N2—N1	173.9 (4)
N2—N1—C2—O1	−15.0 (8)	C3—C4—N2—N1	−5.5 (7)
N2—N1—C2—O2	164.9 (5)	C2—N1—N2—C4	178.4 (5)
O1—C2—O2—C1	2.8 (9)	C8—C9—C10—C5	1.5 (8)
N1—C2—O2—C1	−177.1 (6)	C6—C5—C10—C9	−2.0 (8)
C7—C8—C9—C10	0.2 (8)	C4—C5—C10—C9	175.8 (4)
Br1—C8—C9—C10	179.5 (4)	C8—C7—C6—C5	0.8 (8)
N2—C4—C5—C10	180.0 (4)	C10—C5—C6—C7	0.8 (7)
C3—C4—C5—C10	−0.6 (7)	C4—C5—C6—C7	−177.0 (5)
N2—C4—C5—C6	−2.3 (6)		

### Hydrogen-bond geometry (Å, °)

**Table:**

*D*—H···*A*	*D*—H	H···*A*	*D*···*A*	*D*—H···*A*
N1—H1D···O1^i^	0.76 (10)	2.26 (10)	2.929 (6)	148 (10)
C3—H3A···O1^i^	0.96	2.43	3.242 (8)	142

Symmetry codes: (i) *x*−1/2, −*y*, *z*.

## References

[bb1] Bruker (1997). *SMART* and *SAINT* Bruker AXS Inc., Madison, Wisconsin, USA.

[bb2] Cimerman, Z., Galic, N. & Bosner, B. (1997). *Anal. Chim. Acta*, **343**, 145–153.

[bb3] Flack, H. D. (1983). *Acta Cryst.* A**39**, 876–881.

[bb4] Sheldrick, G. M. (2008). *Acta Cryst.* A**64**, 112–122. 10.1107/S0108767307043930 18156677

